# Do Consumers Want More Nutritional and Health Information on Wine Labels? Insights from the EU and USA

**DOI:** 10.3390/nu8070416

**Published:** 2016-07-07

**Authors:** Azzurra Annunziata, Eugenio Pomarici, Riccardo Vecchio, Angela Mariani

**Affiliations:** 1 Department of Economic and Legal Studies, University of Naples Parthenope, Naples 80133, Italy; mariani@uniparthenope.it; 2 Department of Land, Environment, Agriculture and Forestry, University of Padua, Legnaro 35020, Italy; euguenio.pomarici@unipd.it; 3 Department of Agricultural Sciences, University of Naples Federico II, Portici 80055, Italy; riccardo.vecchio@unina.it

**Keywords:** wine, nutritional labelling, health warnings, cross-country analysis, conjoint analysis, consumer segmentation

## Abstract

The global strategy to reduce the harmful use of alcohol launched in 2010 by the World Health Organization includes, amongst several areas of recommended actions, providing consumer information about, and labelling, alcoholic beverages to indicate alcohol-related harm. Labelling requirements worldwide for alcoholic drinks are currently quite diverse and somewhat limited compared to labelling on food products and on tobacco. In this context, the current paper contributes to the academic and political debate on the inclusion of nutritional and health information on wine labelling, providing some insights into consumer interest in, and preferences for, such information in four core wine-producing and -consuming countries: Italy, France, Spain, and the United States of America. A rating-based conjoint analysis was performed in order to ascertain consumer preferences for different formats of additional information on wine labels, and a segmentation of the sample was performed to determine the existence of homogeneous groups of consumers in relation to the degrees of usefulness attached to the nutritional and health information on wine labels. Our results highlight the interest expressed by European and United States consumers for introducing nutrition and health information on wine labels. However, the results of conjoint analysis show some significant differences among stated preferences of the information delivery modes in different countries. In addition, segmentation analysis reveal the existence of significant differences between consumer groups with respect to their interest in receiving additional information on wine labels. These differences are not only linked to the geographic origin of the consumers, or to socio-demographic variables, but are also related to wine consumption habits, attitudes towards nutritional information, and the degree of involvement with wine. This heterogeneity of consumer preferences indicates a need for a careful consideration of wine labelling regulations and merits further investigation in order to identify labelling guidelines in terms of the message content and presentation method to be used.

## 1. Introduction

Alcohol misuse ranks among the top five risk factors for disease, disability, and death throughout the world and also has serious social and economic consequences for individuals other than the drinker and for society at large [1]. The global strategy to reduce harmful use of alcohol launched in 2010 by the World Health Organization includes, among several areas of recommended actions, providing consumer information about, and labelling, alcoholic beverages to indicate the harm related to alcohol [2]. Labelling requirements on alcoholic drinks worldwide differ greatly [3,4,5] and are generally rather limited in scope compared to labelling on food products and on other products considered damaging to health such as tobacco. While there is a trend globally towards mandatory nutritional information in food product labelling regulation, hailed as an important instrument in promoting healthier eating habits [6], alcoholic beverages are generally exempted from such legislation. For instance, lately in the European Union, according to Regulation (EU) 1169/2011 on the provision of food information, alcoholic beverages have been temporarily exempted from nutritional labelling obligation. Furthermore, unlike what has been done for tobacco labelling, few countries have introduced mandatory warning labels on alcoholic beverages. In the United States, government warning-labels on alcohol containers have been mandatory since 1989. Over the years, other countries have introduced different kinds of mandatory health warnings on alcohol, including South Africa, France, and Germany. Others, such as the United Kingdom and Australia, have chosen to work with the industry to encourage the voluntary placing of consumer information and health warning labels on alcoholic beverages. A recent study revealed that the industry’s commitment to labelling appears not to have been fully met in the UK [7]. Moreover, in Australia, according to the most recent audit, these warnings have still not been adopted on most alcohol product packaging [8], which currently fails to effectively convey health messages to consumers [9].

Finally, initiatives promoted by both consumer and public health organizations in several countries have called for improvements in the labelling of alcoholic drinks. In this regard, the European Alcohol Policy Alliance proposed, in 2012, recommendations for a comprehensive European alcohol strategy including better labelling for alcoholic beverages. This strategy refers in particular to the introduction of an ingredients list, health warnings, and nutritional information [10]. In the USA, in 2003, a proposal for a uniform “Alcohol Facts” label was submitted by the National Consumers League (NCL), the Center for Science in the Public Interest (CSPI), and others in a petition to the Alcohol and Tobacco Tax and Trade Bureau (TTB). The “Alcohol Facts” labels would give consumers clear information about alcohol content, serving sizes, calories, and ingredients [11]. After a decade of lobbying by consumer groups, in 2013, the TTB issued a ruling to allow alcohol beverage makers to voluntarily place serving facts labels on their alcohol-containing beverages [12]. Regardless of the mandatory or voluntary nature of legislation on nutritional and health warning labelling, its effectiveness is closely related to consumer interest in such information and their ability to understand the information included. Consumer interest in nutrition and health-related information on alcohol labelling has been investigated only quite recently, while the effectiveness of warnings has historically received greater attention. As an alcoholic beverage, wine has been largely neglected from this angle, although it has been amply shown that labels constitute a key source of information for wine consumers, providing details on both intrinsic and extrinsic quality cues [13,14,15]. In addition, it should be noted that the bottle of wine and its label are observed and potentially discussed on before, during, and after the consumption. This means that messages transmitted by the label may contribute to the sedimentation of a wider consciousness of people concerning the nutrition and health issues related to wine consumption.

Given this background, the current paper contributes to the academic and political debate on the inclusion of nutritional and health information on wine labelling, providing some insights into consumer support, interest, and preferences regarding such information in four core wine-producing and consuming countries: Italy, France, Spain, and the USA.

## 2. Consumer Attention to Nutrition and Health Labelling: A Brief Review

As widely recognized by the literature, the benefits of nutrition labelling depend on the extent to which consumers’ use of nutritional and health information on labels will shift food consumption towards healthier choices [16]. There is a considerable and varied literature on consumer use and understanding of nutritional and health information in labelling (for a detailed review, see, among others, Campos et al. [17] and Grunert and Wills [18]). A common result of these studies is that the label is a key source of providing nutritional and health information as the consumer’s first exposure to a health-related issue while shopping. Indeed, most research shows that consumers support nutritional labelling initiatives and have positive attitudes towards nutritional labels [18,19,20,21,22].

However, as effectively pointed out by Sørensen et al. [23] current insights into consumers’ attention and use of nutrition and health information in labelling is limited because most studies have used self-administered surveys. It has been observed that, using this kind of methodology, there may be significant overestimation of actual use and understanding of labels [24]. In this regard, for example, it was suggested by Grunert et al. [24] that self-reported behaviour, when compared to measures based on observation and subsequent interviewing on the concrete purchase location, leads to over-reporting by about 50%. This phenomenon is described in the literature as “socially desirable responding” that might lead to such overestimation [25]. In addition, several scholars have underlined the fact that in everyday life, food choices are a question of routine and habit [26]. Thus, consumers cannot and do not process new information to make a thoughtful decision each time they have to buy or choose a food product. 

At the same time, other research has shown that nutritional and health information on labels may often receive scant attention [27,28,29]. This research also highlights the existence of several factors that could limit the consumer’s attention and effective use of information present on the label [30]. Some studies have reported that consumers found difficulties in interpreting nutritional information contained in labels [27,31] and that they express the desire for a simpler presentation of information [32,33]. However, in general, reading labels more frequently is associated with better understanding [17]. Additionally, there is also some evidence that labels are less well understood by people from lower socio-economic groups [27].

Various studies also pointed out that the continuous increase in the amount of information reported on labels, whether mandatory or voluntary, may mislead consumers, especially for food characteristics that are more difficult to understand [23]. Consequently, providing too much information or information that leaves consumers confused may increase the search and information costs on the market.

Time pressure is another factor that limits the effectiveness of labels. Indeed, lack of time is consistently reported as a reason for non-use [27,31]. As most food choices are made very quickly, in a complex environment, it appears natural to assume that a lack of attention could be an important factor limiting the effect of nutrition labels on food choices [32,33,34]. Likewise, other studies show that consumers who spend more time, or report having more time to shop for groceries, were more likely to be label users [35]. As reported by Sørensen et al. [23] the conflict between information overload and the limited time to process information in real purchase situations leads to a reduction in consumers’ attention level with counterproductive effects. Consumers may get confused or misinterpret information, making an inappropriate purchase decision, or they may decide to completely ignore labelling, becoming immune to the information presented [36].

A number of studies also suggest that the label format and content could limit the consumer’s attention and use of nutritional and health information in labels [17]. Previous research has shown that consumers prefer short front-label claims to lengthy back-label explanations, or a combination of both (e.g., [18,37]). Furthermore, studies have reported greater effectiveness for labels using graphics and symbols, adjective labels, and labels with minimal numerical content [22,38].

Previous studies have also shown that attention to, and use of, nutrition and health information on the label can vary significantly, even with respect to the category of product [18]. In this regard, a very limited number of studies have analysed consumer interest in nutritional labels on alcoholic beverages, revealing some common findings [39,40,41,42,43]. In particular, these studies bring to light a clear information gap (a limited knowledge of the nutritional content of alcoholic drinks) and the strong interest expressed by consumers for the inclusion of nutritional information on the label of alcohol beverages. This evidence was also confirmed in wine-specific studies [44,45]. At the same time, some unexpected consequences from information disclosure have been highlighted. Paradoxically, consumers who have an incorrect perception (overestimated) of the calories, fat, and carbohydrates of an alcoholic drink, thanks to the availability of serving facts information, could increase consumption [40].

With regard to alcohol warning labels, there is broad consensus in the literature that this kind of information may improve knowledge, raise awareness, and prompt discussion on the harmful health and social consequences of alcohol abuse. Furthermore, no negative effects have been demonstrated [3,45,46,47,48,49,50]. Nevertheless, evidence on the impact of drinking behaviour is controversial. The low effectiveness seems mainly related to some features related to the warning currently implemented, such as its weak content, poor visibility, and the lack of pictorial content to illustrate the consequences of alcohol abuse [51].

## 3. Materials and Methods

A cross-country survey was carried out in Europe (France, Spain, and Italy) and the US. The questionnaire used for the survey consisted of five parts. These parts analysed the following: consumer wine-related behaviour (e.g., drinking frequencies and place of consumption); label use and label belief (with reference to both food products in general and wine); knowledge of nutritional aspects of wine consumption and interest in nutritional information and health warnings on wine labels; and socio-demographic characteristics. In the final part of the questionnaire, consumer preferences for different formats of additional information on wine labels were investigated by presenting ten different wine back label profiles, with picture cards based on a conjoint design. Some questions and picture cards used were adapted to better reflect differences in current labelling legislation between Europe and the USA.

**Figure 1:**
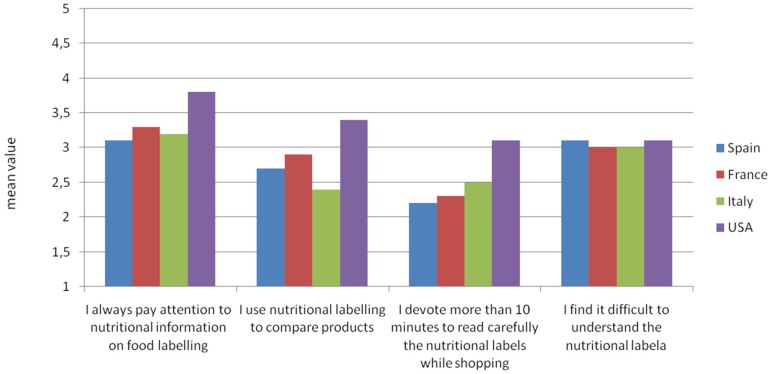
To what extent do the following statements reflect your everyday food shopping behaviour (mean scores related to scale ranging from 1 = not at all, to 5 = completely)?

Wine-related behaviour was assessed using questions previously validated in studies on wine purchasing behaviour [15,52]. Statements used to evaluate consumer label use and label beliefs were adapted on the basis of previous research [16,53,54] and reported in Figure 1.

In Europe, data were collected from January to June 2015 using an online survey platform. Prior to the final administration, the questionnaire was validated using 40 consumers, 10 for each country, checking for respondents’ full understanding and analysing optimal interview length with the support of a professional recruiting company. The European target population was specified as individuals between 18 and 70 years old, drinking wine at least once a month. In the US, respondents were selected by a professional market research agency from a representative online panel in August 2015. The US target population was specified as individuals between 21 and 70 years old, drinking wine at least once a month. A total of 1016 valid interviews were carried out, broken down as follows: 330 in Italy, 185 in France, 195 in Spain, and 306 in USA (east coast).

The generated data were analysed twice. In the first phase, a descriptive analysis was performed through the frequency procedure, providing basic statistics and graphical displays useful for describing many types of variables. Moreover, the cross-tab procedure forms (two-way and multi-way tables) were useful for finding shared relations between the variables and providing a variety of tests and measures of association for the two-way tables. Rating-based conjoint analysis was then applied to verify consumer preferences for different formats of additional information on wine labels. Conjoint analysis is based on a main effects analysis-of-variance model. Subjects provide data about their preferences for hypothetical products defined by specific attribute combinations. It decomposes the judgment data into components, based on qualitative attributes of the products. A numerical part-worth utility value is computed for each level of each attribute. Large part-worth utilities are assigned to the most preferred levels, and small part-worth utilities are assigned to the least preferred levels [55]. The attributes with the largest part-worth utility range are considered the most important in predicting preference.

Based on previous studies, the conjoint design consisted of four product attributes: price (average market price; average market price plus 10%); health warnings (not present; logo + warning; only logo); nutritional information (not present; glass with kcal indication; nutritional panel); and servings per container with indications of units recommended not to exceed (present; not present). The choice of levels related to health warnings, nutritional information, and servings per container was based on the relevant literature related to general consumer preferences for food nutritional labelling, e.g., [53,56,57,58] and consumer preferences and attitudes towards wine labelling, e.g., [42,59]. The decision to include price in the conjoint design was derived from previous studies that found that price was a key driver of wine choice [14,60].

It should be highlighted that, in the US market, the relative levels of health warning attributes differ from those in Europe. In the US, alcoholic beverages have to report the following warning statement: (1) According to the Surgeon General, women should not drink alcoholic beverages during pregnancy because of the risk of birth defects; and (2) Consumption of alcoholic beverages impairs your ability to drive a car or operate machinery and may cause health problems. Thus, only for the US market conjoint design were the relative levels of this attribute present in the form of a government warning, in the simplified form with a logo, or in the form of a logo with claims. Even the price levels proposed to respondents were on average higher than those in European markets, taking into account average actual national retail prices. Even though there are small differences between the levels of health warnings and price attributes, the conjoint design is unaffected by such differences because the ratio of the number of levels is maintained.

Based on these attributes and levels in our analysis, the consumer choice model is specified as follows:

*Pi* = β*i*1 + β*i*2 PRICE + β*i*3 HEALTHWARN + β*i*4 NUTINFO + β*i*5 SERVING + ε*i*, *i* = 1,…, *I*,

where *P* is the preference rating of the hypothetical wine for the *i*th individual. PRICE is a variable for price levels. HEALTHWARN is the variable for health warning levels. NUTINFO is a variable for the nutritional information content. SERVING is a variable for the units per container with indications of units recommended not to exceed, and ε is a random error term. Interviewees were asked to express their preferences for each card shown, using a metric preference scale from 1 = not preferable at all to 5 = totally preferable. Conjoint part-worth utilities were estimated using ordinary least squares regression (OLS). Mean differences between part-worth utilities were investigated using ANOVAs with Tukey tests. Furthermore, Kendall’s b-tau and Tukey’s tests were performed on utility scores to evaluate significant differences between countries. Measures of design efficiency were calculated prior to data collection and proved to be very good compared to the optimal design (in terms of A-efficiency, D-efficiency, and G-efficiency).

## 4. Results

### 4.1. Socio-Demographic Characteristics and Wine-Related Behaviour

The socio-demographic characteristics, wine drinking, and purchasing habits of the sample are summarised for each country in Table 1. With reference to gender and age, there are no significant differences among countries. Considering the presence of medical disorders that could influence food choices, it should be noted that there are substantial differences. The French show a higher incidence of individuals without health disorders (45% of cases), whilst, for US consumers, this incidence is very low (8% of cases). In particular, in the USA, the number of individuals with cardiovascular problems and who are affected by obesity/overweight is 28% and 32% respectively, higher than in European countries.

Analysing the variables related to wine-drinking frequencies, the results show that among Europeans, French consumers reported a higher frequency of consumption (26% every day and 32% 3–4 times a week), followed by the Spanish (24% every day and 28% 3–4 times a week), while the Italians, on average, drink wine less frequently: 18% of Italians drink wine every day, another 18% 1–2 times per month, and 13% rarely. Compared to European consumers, Americans stated that they drink less: 15% rarely (once a month) and 24% 1–2 times a month. Although our sample is not representative in terms of national wine consumption frequency, our results roughly reflect the frequency of consumption recorded in national surveys of each country. In Italy, the wine consumption data emerging from our sample are in line with the latest figures published by Italian National Institute of Statistics (ISTAT) [61]: about 20% of Italians state a daily wine consumption (consuming on average one or two glasses per day), while the remaining population consumes wine more sporadically. In Spain, a report published by the Spanish Observatory of wine market (OEMV) [62] showed that almost 28% of participants had a daily consumption frequency of wine, and 25% of the sample two or three times a week, while 8% once a month. In France, FranceAgriMer [63] reported that around 16% of those surveyed use to drink wine daily, while occasional drinkers (between one and four times a week) account for about 60% of the sample. For the US, a recent research from the Wine Market Council [64] showed that of the 230 million adults, 40% drink wine. Of these, 33% are defined as high frequency drinkers, or those who consume wine more than once a week, and the remaining 67% are considered occasional drinkers, as they drink wine once a week or less.

Consumers from all countries reported drinking on average two glasses per occasion, even if the proportion of those who claimed to drink three glasses on each occasion is much higher in France (24%) than in Italy and the USA (both 11%), and somewhat higher than in Spain (18%).

### 4.2. Label Use and Label Beliefs

Label use and label beliefs were first analysed using five statements related to nutritional labelling on food, asking respondents to what extent specific statements reflected their everyday food shopping behaviour (scale ranging from 1 = not at all to 5 = completely). Figure 1 shows the mean value of the degree of agreement expressed by respondents. On average, consumers of all countries stated that they pay attention to nutritional labels when buying food. However, some differences were reported in the use of such information and in the amount of time spent reading nutritional labels when shopping. US consumers on average tended to pay more attention to nutritional labels compared to consumers in other countries. As reported in Figure 1, for US consumers the mean value of agreement with the first statement is 3.8 (SD 0.93), while it is lower for Italian (3.2, SD 1.16), French (3.3, SD 0.95), and Spanish (3.1, SD 0.93) customers. Furthermore, US consumers tend to use this information more to compare food products (with a mean value of 3.4, SD 1.13) than Italian (2.4, SD 1.01), French (2.9, SD 1.09) and Spanish (2.7, SD 1.18) customers. Obviously, this could be due to the mandatory introduction of panel facts since the 1990s. Furthermore, it should be noted that consumers’ actual attentiveness to labels and nutritional information on food and wine is generally considered to be lower than what consumers self-report [14,24]. Thus, the previously discussed results could represent an overestimation of actual buying behaviour while shopping.

The most interesting aspect emerging from this part of the analysis is that, in all four countries, respondents stated that they had some difficulty understanding the information on nutritional labels. In this regard, our results report a mean value of 3.0 in both France (SD 1.23) and Italy (SD 1.31) and a mean value of 3.1 in both Spain (SD 1.17) and USA (SD 1.22).

Consumers were also asked to indicate whether they changed habits on the basis of the information in the nutritional label of food and beverages. In European countries, the percentage of those who stated that they often changed their habits after reading nutritional labels lies at 15% or below (10% in Italy, 11% in Spain, and 15% in France). By contrast, the percentage of those who claimed that they never changed their habits exceeds 20% in all countries (29% in Italy, 24% in Spain, and 22% in France). In the USA, about 24% of respondents said that they often changed, while 40% said that they seldom do.

The survey then focused specifically on wine labelling, asking consumers to indicate first their reading frequencies of the front label and then of the back label when choosing wine. With reference to the front label, French consumers stated they always read it in 54% of cases. In Spain, respondents who always read front labels was 44%, and in Italy, 40%. Moreover, Italians show a higher percentage of individuals who stated they do not read them at all (6%) or only on the first purchase (12%). As for US consumers, the proportion of those who claim they always read the information on wine front labels is lower than the European average (25%).

However, if we consider the back label, the incidence of individuals who always read this label tended to decrease in all four countries. Indeed, more than 40% of French and Spanish consumers stated that they always read the wine back label, while, for Italians, this percentage drops to 26%, and, for the USA, it is as low as 18%.

In order to investigate what type of wine label information consumers look for, the interviewees were asked to express the degree of importance attached to a set of proposed information (using a five-point scale from 1 = not at all to 5 = extremely). As reported in Table 2, the information generally considered the most important by European consumers (i.e., which obtained a higher average score) comprises the area of origin and the presence of designation of origin. For these two attributes, the degree of importance assessed by Italian, French, and Spanish consumers is almost identical.

Italians are those who paid greater attention on average to alcohol content and to suggestions for consumption, while both the Spanish and Italians attached more importance to sensory description than French consumers. US consumers attached greater importance to information regarding the manufacturer’s name, grape variety, and vintage, which were considered less important especially in Italy and Spain. US consumers also attached greater importance to information on prizes and awards obtained by the wine. The existence of such differences in the perception of the importance of the information on the label clearly reflects the differences in consumption preferences of individuals from different geographic areas. Finally, it should be highlighted that consumers from all countries attached less importance both to the indication of the company website and to the presence of allergens or sulphites.

### 4.3. Knowledge and Interest in Nutritional and Health Information on Wine Labels

In order to determine the respondents’ knowledge of wine nutritional properties, individuals were asked to indicate how many kcal were roughly contained in a glass of red wine of medium alcohol content (125 mL, 13% volume). Findings show significant differences (chi-square; *p* < 0.005) in the level of knowledge of the amount of kcal in a glass of red wine among countries. Among Italian consumers, only 22% were aware of the kcal amount in a glass of wine. Indeed, most individuals tended to underestimate the content of kcal (51%), or even consider that wine has no kcal at all (12%). Similarly, in Spain, the percentage of those who tended to underestimate kcal content in wine is high (50%). However, the correct answer was indicated by more than 30% of the sample. By contrast, in France, consumers showed a greater knowledge of kcal contained in a glass of wine (36%) and tended to underestimate the calorie content. Among US consumers, 28% were aware of the kcal in a glass of wine, while 43% tended to underestimate kcal content and 29% overestimate it.

**Figure 2:**
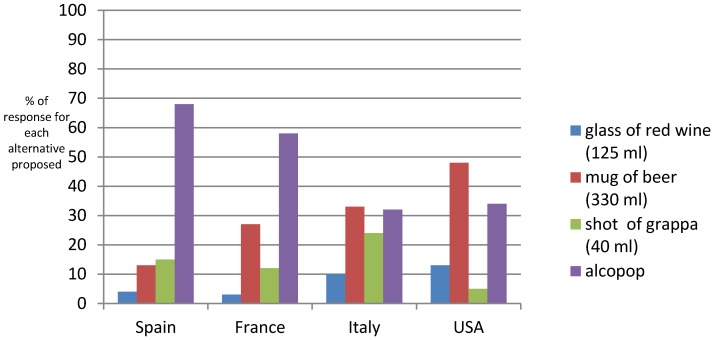
Consumer responses vis-à-vis alcoholic drinks with the highest kcal content (%).

Consumers were also asked to indicate which alcoholic drinks contained more kcal, choosing between a glass of red wine, a mug of 330 mL of beer, an alcopop, and a shot of grappa (or spirits). As reported in Figure 2, in France and Spain, consumers indicated the correct option (alcopop) in most cases (respectively, 58% and 68%). By contrast, Italian and US consumers indicated the correct option only in 34% of cases, assigning a greater kcal content to a mug of beer (in 48% of cases in the US and 33% in Italy).

**Figure 3:**
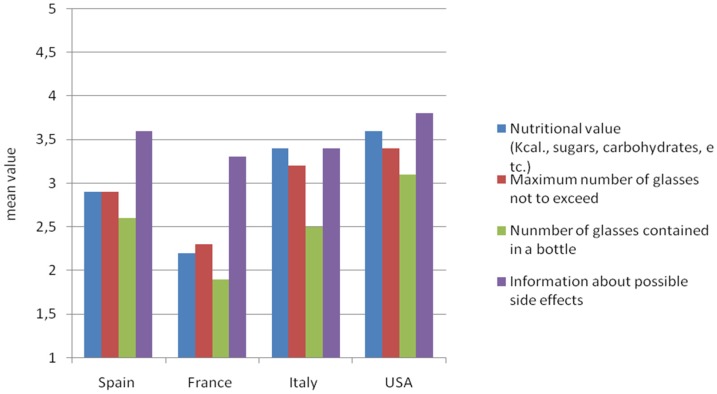
How interested are you in receiving the following information on wine labels? Mean value on a five-point scale ranging from 1 = not at all, to 5 = totally.

Respondents were then asked to indicate their interest in receiving additional information on wine labels with specific reference to a set of information (using a five-point scale from 1 = not at all to 5 = extremely). Figure 3 reports results as mean values. Consumers from all countries stated a higher interest in information on possible side effects related to excessive consumption, whereas they showed lower interest in the number of glasses contained in a bottle. Moreover, findings underline significant differences among countries.

American and Italian respondents seemed more interested in receiving additional information on wine nutritional value than French and Spanish consumers. In this regard, American and Italian respondents expressed a mean interest respectively of 3.6 (SD 1.32) and 3.4 (SD 1.16), versus 2.2 (SD 1.09) and 2.9 (SD 1.21) of French and Spanish (*p* < 0.001). French consumers are those who assigned the lowest score to nutritional value (mean value 2.2, SD 0.83), giving higher scores to the maximum amounts not to exceed the recommended limits of alcohol consumption (mean value 2.3, SD 0.87). With regard to Spanish respondents, the score for nutritional value information is close to the overall sample average and is equal to maximum amounts not to exceed the recommended limits of alcohol consumption (mean value 2.9, SD 0.92).

Finally, respondents were presented with five different warning statements and asked to express how useful they consider such statements in their choices (based on a five-point scale from 1 = not at all, to 5 = extremely). As reported in Table 3, consumers from all countries tended to assign a high utility score to “ban on alcoholic beverages to children under 18/21 years” and “do not drive after drinking”. For these two warnings, the scores tended to converge, exceeding 4.1 in each country. Nevertheless, it is worth noting that significant differences (*p* < 0.001) emerge for the statement “avoid drinking alcohol during pregnancy”. This warning indeed was considered, on average, more useful by US and French consumers, but was less valued by Spanish and Italians. Moreover, for the warning “avoid drinking alcohol when you are taking medicines”, significant differences emerged, revealing a higher utility of American consumers. Similar outcomes were found for the statement “alcohol increases the risk of violence”, albeit with a lower significance level.

## 5. Conjoint Analysis of Different Formats of Additional Information on Wine Labels

**Figure 4:**
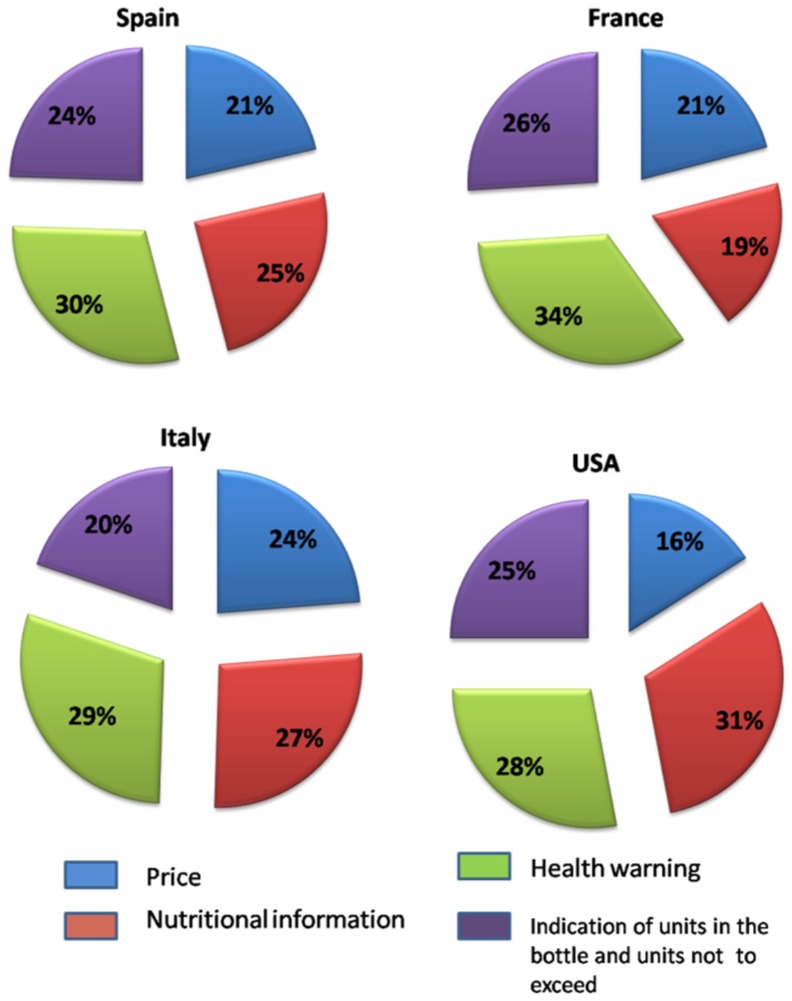
Mean relative importance assessed for each attribute in each country.

**Figure 5:**
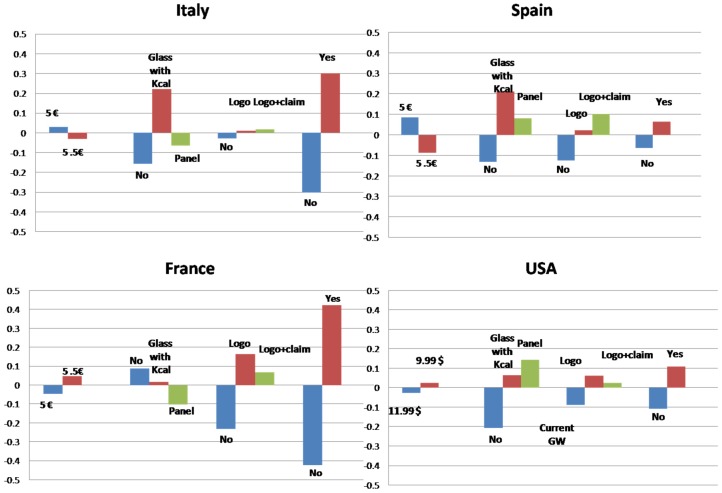
Part-worth utility assessed for each level in each country.

Conjoint results confirm the general interest of respondents in nutritional information and health warnings on wine labels. However, significant differences with the Bonferroni test (*p* < 0.005) between countries emerged among the part-worth utility values assigned by consumers to different pieces of information. The mean relative importance assessed for each attribute and part-worth utilities are summarised in Figure 4 and Figure 5. Goodness-of-fit of conjoint analysis is confirmed by the high values for Pearson’s R and Kendall’s Tau-b statistics for each country.

With regard to the mean relative importance of each attribute (Figure 4), it should be noted that the presence of a health warning is the attribute to which consumer’s assigned greatest utility in European countries. Conversely, US consumers assigned greatest utility to nutritional information (31%). Among European consumers, Italians attached more utility to nutritional information (27%) on wine labels, followed by the Spanish (25%). The French, instead, considered this information least useful (19%), attaching more utility to health warnings (34%), followed by the indication of the units in the bottle and glasses not to exceed (26%) and price (21%). By contrast, for Italian consumers, the indication of the units in the bottle and glasses not to exceed was considered the least useful.

The part-worth utility assessed for each level of attributes in each country is reported in Figure 5, revealing some indications as to how consumers preferred to receive this information on the label. With reference to nutritional information, Italian and Spanish consumers preferred the simplified version of a glass with only kcal. As regards health warnings, greater utility was assigned to the full version with a logo and claim. French consumers instead attached more utility to the version without nutritional information, expressing a preference for the health warning with only a logo. US consumers preferred the detailed version of nutritional information (with a nutritional panel). While with reference to the health warning, they tended to attach more utility to the version with only the logo instead of the current one (with only the government warning).

It is also important to underline that, in all four countries, consumers expressed a positive utility for the presence of the number of units not to be exceeded on the wine label.

With reference to price, while Italian and Spanish consumers preferred the average market price level, French and US consumers tended to prefer a higher level of price. However, it should be noted that the relative difference in the two prices used, i.e., average market price and average market +10%, did not differ enough to affect choice.

## 6. Segmentation of the Total Sample

**Figure 6:**
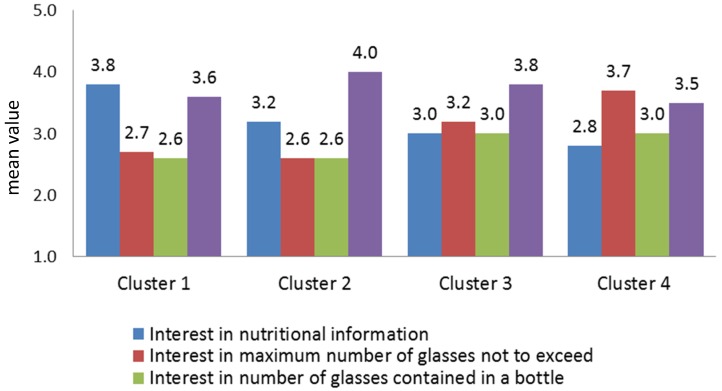
Cluster profile in relation to interest in additional information on wine labels.

The total sample was segmented using cluster analysis to determine the existence of homogeneous groups of consumers. Variables used for clustering were related to the degree of interest in additional information on wine labels (reported in Figure 6). One-way ANOVA, with a comparison of mean values for other variables related to the use and familiarity of information provided on wine labels, attitudes towards nutritional labelling of food and knowledge of nutritional properties of wine, and cross-tabulation with chi-square statistics for socio-demographics characteristics, was also performed in order to profile the clusters. Non-hierarchical clustering, with k-means cluster analysis, was performed to obtain the segments. From the application of this method, it was found that the division into four groups was the ideal solution, where homogeneity is maximised within the individual clusters and minimised between them.

As shown in Figure 6, the identified clusters differ significantly in relation to the degrees of usefulness attached to the proposed additional information on wine labels (nutritional value, health warning and indication of number of glasses not to exceed, and number of glasses contained in a bottle). Furthermore, the univariate ANOVA showed the existence of significant differences between the groups in relation to variables linked to the use and familiarity of information provided on wine labels.

The first cluster consisted of 22% of the total sample and included individuals who preferred to see nutritional information on wine labels, followed by health warnings. This group also pays greater attention to the information presented on front wine labels as well as that on the back label. Additionally, this segment attached greater importance than the sample average to the information related to the indication of allergenic substances and alcohol content on wine labels.

With regard to the general attitudes towards nutritional labelling of food, these consumers showed a stronger attitude towards nutritional labels, claiming that they read them more frequently and easily understood their content. Knowledge of the nutritional properties of the wine in this cluster was also higher than the sample average, including a greater number of individuals who correctly answered the questions about the caloric content of wine. With reference to socio-demographic variables, this cluster mainly comprised middle-aged women (61% between 35 and 55 years) with a higher level of education. Finally, even if the geographical origin is not statistically significant, it should be pointed out that, in this group, there was a higher concentration of US consumers.

The second cluster, distinguished for being the largest (35% of respondents), tended to attach higher utility to health warnings, followed by nutritional information. This cluster consisted mainly of individuals who read the information on the front wine label more frequently, whereas, for the back label, the reading frequency is more sporadic compared to Cluster 1, but higher than Clusters 3 and 4. With respect to the attitude towards nutrition labels, these consumers stated that they often read this information while shopping but tended not to use it to compare products and considered the information too complex to understand. From a socio-demographic perspective, this cluster included mostly young women (43% are below the age of 45) and had a higher concentration of individuals with children under 16 years in the household.

The third cluster (28% of the sample) attached great value to health warnings, followed by the indication of the suggested number of units not to exceed with consumption. These consumers assigned less importance to nutritional information compared to Clusters 1 and 2, as confirmed by their general attitude towards nutritional labelling of food. This cluster groups individuals who read nutritional labels mainly only upon first purchase and who devote little time to reading nutritional labelling while shopping. As regards knowledge of the wine nutritional aspects, this group tended to overestimate the kcal content of a glass of wine. Furthermore, these consumers also read the front and back label information less frequently and consume more wine than the other two clusters. From a socio-demographic perspective, this cluster included mostly adult men (62% over 35 years) with an average level of education.

The fourth and final cluster is numerically the smallest, including 15% of respondents, and stands out for attaching greater value to the indication of units not to exceed, followed by the health warning. These consumers showed a poor attitude towards nutritional labels due to the prevalence in this group of individuals who stated that they never read them. At the same time, consumers in this cluster generally knew little about the nutritional properties of wine and tended to underestimate the calorie content. Moreover, in this case, as in Cluster 3, the consumption of wine is more frequent than in Clusters 1 and 2, highlighting the fact that consumers who claim a higher frequency of consumption are often the ones who attach less importance to the nutritional information or to health warnings.

## 7. Discussion

The results from the current study work to fill the literature gap concerning consumer interest in nutritional and health warnings on wine labels and consumer preferences towards different ways of conveying such information on labels. Our results also show the existence of a demand for information concerning nutrition and health issues related to wine consumption. Furthermore, our findings highlight some significant differences among the stated preferences of European and US consumers towards the information-delivery modes.

Overall, for food and beverages, our findings reveal that, although both European and US consumers stated that they pay attention to nutritional labels when buying food, they have some difficulty understanding the information. It is also worth pointing out that, in many cases, our results show that nutritional information on food labels did not appear to have had any impact on changes in consumer behaviour, especially among European respondents. In addition, respondents from each country stated that they do not devote much time to carefully reading nutritional labels when food shopping. These results are consistent with other research on information on food labels conducted both in Europe and the USA [18,27,30,65], wherein nutrition labelling was reported to be particularly confusing for consumers, especially due to the use of technical, scientific, or numerical information. Previous research has highlighted this divergence between consumers’ stated interest in nutritional labelling and the actual use of such information in both Europe and the US, suggesting that a lack of use is a question not only of understanding, but also motivation, e.g., [18,30,65,66].

With specific reference to wine labels, it was found that consumers, especially in Europe, stated a high level of use, confirming the central role of labels in influencing wine choices [13,14,15]. However, our results revealed a greater interest of consumers in the information on the front rather than the back label. This consideration suggests that the positioning of the information is a crucial element in influencing purchasing decisions, as highlighted by previous research [67,68].

With regard to wine nutritional properties, our results revealed that both European and US consumers display a knowledge gap of the calorie content of wine. In many cases, such content was underestimated by respondents, especially by Italian and Spanish consumers. In addition, consumers from all countries had difficulty evaluating the kcal content of the different types of alcoholic beverages. In particular, Italian and US consumers tended to assign a higher caloric content to a glass of wine than other drinks. This knowledge gap confirms findings from previous research [41,43]. In this regard, Wright and colleagues [41] showed that the actual knowledge of nutritional information not only influences consumer perceptions of the healthiness of different beverages but also affects their choices among different alcoholic beverages. By contrast, Dale and colleagues [69] in a recent study indicated that actual knowledge of the nutritional value of alcoholic beverages with nutritional labels would not affect the drinking habits of US students.

The most interesting result that emerges from conjoint analysis is that interest expressed in receiving nutritional information on wine labels differs according to the country concerned. With reference to the modes and formats of additional information, European consumers revealed a preference for a simplified version of nutritional information, such as an image of with an indication of kcal. Conversely, US consumers preferred the standard nutritional panel. Moreover, in the case of health warnings, conjoint results revealed that the majority of Italian and Spanish consumers preferred the full version (logo with statement), while the French and US preferred the simplified version only with a logo.

In addition, results from the segmentation demonstrate the existence of significant differences between consumer groups with respect to their interest in receiving additional information on wine labels. These differences are not only linked to the geographic origin of the consumers or to socio-demographic variables, but are also related to their wine consumption habits, their attitude towards nutritional information, and their degree of involvement with wine. This is in concert with other research that suggested that consumers tend to evaluate labels differently in relation to their wine-related behaviour and not only by country [70,71]. In addition, segmentation analysis highlights the fact that consumers who claim a higher frequency of consumption are often the ones who attach less importance to the nutritional information or to health warnings.

Therefore, it may be stated that, even if our results reveal consumer interest in nutritional labelling on wine bottles, different levels of interest and the perceived usefulness by consumers indicate a need for the careful consideration of wine labelling regulations. Finally, with specific reference to wine attributes, it should also be considered that, as suggested by Mueller et al. [72], direct measurements of packaging attribute importance may not reveal true preferences. This would suggest considerable caution in using direct importance measures.

## 8. Conclusions

Despite their proven negative impact on health and society arising from excess consumption or abuse, alcoholic beverages have benefited from special treatment. At present, alcoholic beverages are exempted from international conventions governing all other psychoactive substances and from key food legislation (no labelling of ingredients or nutritional information). As discussed by Rehm et al. [73], the reason why alcoholic beverages are treated so exceptionally is subject to debate and, even if currently not fully understood, the lack of information about alcohol-attributable risks seems to play an important role. Among the various tools that should be implemented to raise awareness about the risk of alcohol abuse within a comprehensive strategy of public drinking education, several consumer and public health organizations (such as the WHO) have long been calling for mandatory proper content and nutritional labelling, including warnings regarding health and social risk. This issue is very controversial, as policymakers must weigh the benefits and costs of labelling as well as the distribution of benefits and costs to determine whether mandatory labelling is an appropriate policy option, as exemplified by Magat and Viscusi [74].

The effectiveness of labelling and warnings in educating consumers and in changing unhealthy drinking behaviour is debated in the literature and should be fully explored. Furthermore, introducing mandatory labelling requirements generates a variety of costs and difficulties in implementation, especially for small businesses and small wineries [75]. Considering the general interest expressed by consumers in including nutritional and health information on wine labels, both the wine industry and public bodies should cooperate, trying to minimise the costs for producers and maximising the effectiveness of messages to consumers, avoiding the risks of misinterpretation. In this regard, it should be noted that some alcoholic drink manufacturers and winery associations have already begun to voluntarily provide nutritional information or information about the risks of harmful consumption to their customers and are co-operating with public institutions to find the most effective ways of providing this information to consumers.

The results herein offer further insights into consumer perspectives. It specifically emerged that consumers support additional nutritional and health information on wine labels, while significant differences were found among stated preferences of information delivery modes in different countries. It should be stressed that delivery modes matter: for instance, the simplified nutritional label solution (glass with a rough kcal estimate) preferred by European consumers could be more cost-effective, being easier to implement, while avoiding companies that promote wine as a product with zero fat. Overall, this heterogeneity of consumer preferences needs further investigation in order to draw up labelling guidelines in terms of message content, the method of presentation, label size, and position [43].

Further research should aim to overcome the main limitations of the current results. First, sampling issues, with reference to size and composition, limit the representativeness of our results. Furthermore, our findings were based on the self-reported use of labelling, which is believed to lead to considerable over-reporting [18,23,24,30]. In addition, as reported by Lockshin and Corsi [76], differences in importance attached to wine label information also stem from the way in which the research question is posed, and direct response surveys are not always able to measure subconscious influences on preference or choice. Therefore, new research avenues should focus on alternative approaches to analysing the natural behaviour of consumers, using, for example, eye-tracking methodology that allows participants’ visual attention towards specific areas of interest to be tracked.

## Figures and Tables

**Table 1:** Sample socio-demographic characteristics, wine drinking, and purchasing habits (%).

	Category	Italy	Spain	France	USA
Gender	Female	51	52	50	50
Age *	35–44	22	22	21	24
	45–54	21	23	22	25
Education	Bachelor’s degree	31	43	40	39
	High school diploma	43	33	36	24
Income	Medium	68	66	63	63
Medical disorders that influence food choices	No disorder	36	32	45	8
	Cardio-vascular problems	22	18	16	28
	Obesity/overweight	15	24	12	32
Wine-drinking frequencies	Every day	18	24	26	16
	3–4 times a week	20	28	32	19
	1–2 times a week	31	25	28	28
	Twice a month	18	15	10	24
	Rarely (once a month)	13	8	4	15
Number of glasses per occasion	1	41	30	12	20
	2	40	42	43	51
	3	11	18	24	11
	>3	8	10	21	18

* For the US population, this age range is between 21 and 24.

**Table 2:** Degree of importance attached to a set of proposed information reported in wine label mean value and standard deviation.

	Spain	France	Italy	USA
Manufacturer name	3.51 (0.81)	3.85 (0.82)	3.59 (0.76)	4.3 (0.60)
Grape variety	3.13 (0.90)	3.46 (1.01)	3.07 (0.85)	4.2 (0.81)
Vintage	3.01 (0.90)	3.61 (0.87)	2.86 (1.01)	3.9 (0.89)
Prizes/Awards	2.54 (1.01)	2.6 (0.96)	2.38 (1.01)	3.2 (0.91)
Area of origin	4.01 (0.84)	3.95 (1.05)	3.85 (1.02)	3.7 (1.03)
Designation of origin (e.g., PDO, PGI)	3.86 (1.03)	3.84 (1.06)	3.81 (1.03)	2.6 (1.04)
Suggestions for consumption	2.31 (1.02)	2.17 (1.01)	2.67 (1.09)	2.9 (1.02)
Alcohol content	2.53 (1.18)	2.22 (1.22)	3.15 (1.26)	2.8 (1.04)
Presence of sulphites or allergens	2.04 (1.13)	1.98 (1.21)	2.33 (1.01)	2.4 (1.10)
Sensory description	2.89 (1.11)	2.07 (0.87)	2.94 (1.01)	2.7 (1.03)
Winery website	2.16 (0.98)	2.27 (1.05)	1.89 (1.08)	2 (1.12)

PDO: Protected Designation of Origin; PGI: Protected Geographical Indication.

**Table 3:** Perceived utility of warnings *.

	Italy	Spain	France	USA	Significance **
Ban on alcoholic beverages to children under 18 ***	4.2	4.2	4.1	4.1	0.108
Do not drive after drinking	4.3	4.2	4.4	4.4	0.266
Avoid drinking alcohol during pregnancy	3.6	3.8	4.2	4.4	0.001
Avoid drinking alcohol when you are taking medicine	3.9	4	3.9	4.3	0.009
Alcohol increases the risk of violence	3.8	3.9	3.7	4.1	0.041

* Based on a five-point scale from 1 = strongly useless to 5 = strongly useful; ** Statistical test: F-test; *** 21 years for the US market.

## Abbreviations

The following abbreviations are used in this manuscript:

WHO	World Health Organization
EUROCARE	European Alcohol Policy Alliance
EUFIC	European Food Information Council
NCL	National Consumers League
CSPI	Center for Science in the Public Interest
TTB	Tobacco Tax and Trade Bureau
